# Effects of Free Sugars Intake on Human Health and Disease

**DOI:** 10.3390/nu16010031

**Published:** 2023-12-21

**Authors:** Pauline Emmett

**Affiliations:** Centre for Academic Child Health, Bristol Medical School, University of Bristol, Bristol BS8 1NU, UK; p.m.emmett@bristol.ac.uk

Calling all researchers with data on dietary intake to consider investigating dietary sugars (added or free sugars) in relation to nutrients and food groups or food classifications. Sugar is not consumed in isolation, and I have made a case below that the consumption of free sugars, which for the most part means added sugars, has a profound affect on the balance of the human diet. Your data can help to provide evidence of that unbalanced diet and how it relates to poor health outcomes.

I suggest that it is misleading to study ‘free sugars’ in isolation because they are mostly found in combination with other ingredients in the human diet. The liking for sweet tastes is an innate characteristic of humans [1] which can be easily exploited to encourage the excess consumption of sweet foods and drinks. Free sugars are very often combined with other nutrients such as starch and fat or with other tastes such as sourness. The sweetness of free sugars is what makes these foods and drinks, e.g., cookies (sweet biscuits), chocolate bars, cakes and sugar sweetened beverages (SSB), including carbonated SSB, highly palatable. These foods and drinks are widely available, often have a long shelf-life and are strongly advertised and very profitable. To clarify, fruit and vegetable juices, which are naturally sweet, are often considered to be healthy alternatives to SSB; however, they are classified as a source of free sugars. They are not classified as added sugars, and this is the main difference between free and added sugars.

A cross-sectional investigation of the diets of British adults looked at dietary fat in relation to free sugars intake (in foods only) using weighed food records and food frequency questionnaires (FFQ). In this study, free sugars were assessed as non-milk extrinsic sugars (NMES), which are almost identical to free sugars (within 2%). The study found that as free sugars intake increased, so did dietary fat intake, and that half of this increase was in sweetened fat-containing products, e.g., cookies, cakes, chocolate, etc. [2]. Furthermore, as free sugars intake increased, the amount of fruit eaten decreased. Similarly, in Australian adults, those with high free sugars intake ate less fruit and vegetables and more discretionary foods than those consuming less free sugars [3]. Thus, the diets eaten in both these countries by the high free sugars consumers were of a lower quality than those of the low free sugars groups.

The recommendation regarding free sugars intake in the UK has been revised downwards from <10% to <5% of energy from free sugars [4]. In the British National Diet and Nutrition Survey (NDNS), the mean intakes of free sugars were 11.8, 14.7 and 15.4% of energy in children in the age groups 1.5–3, 4–10 and 11–18 years, respectively. Very few children had a free sugars intake of <5% of energy, and those consuming >13% energy had a lower diet quality than those consuming <10% free sugars [5]. In Germany, in the Donald study, children’s diets have been studied continuously since 1985, recruiting new children each year and following them as they grow. German children have an intake of around 16 to 17% energy from free sugars, with 5% of that coming from a food group covering sugar, sweets, chocolates, jelly and ice cream. The Donald study has not, so far, published data on the quality of the diet, or on the amounts of fruits and vegetables consumed in relation to free sugars intakes [6]; I encourage them to do so.

A study that has investigated diet quality in relation to high free sugars intake was carried out with children aged 7, 10 and 13 years in a UK longitudinal birth cohort study (ALSPAC). Two different dietary patterns were obtained using reduced rank regression: one high in free sugars, dietary fat and energy density but low in fibre, and the other high in free sugars but not high in fat or energy density [7]. The first pattern was longitudinally associated with increasing adiposity in the children, while the second pattern was not. This suggests that fat, fibre and free sugars intake in combination are important for obesity development. It is noteworthy that the high fat and free sugars pattern was associated with low fresh fruit intake, while the high free sugars pattern was not [7]. The food groups that contributed most to high scores in the pattern that was high in free sugars, dietary fat and energy density but low in fibre were confectionery and chocolate, cakes and cookies, sugary drinks (SSB), low fibre breads and crisps (chips). The foods that were least consumed in this dietary pattern were fresh fruit, vegetables (not fried), high-fibre breakfast cereals, high-fibre bread and potatoes (boiled). This dietary pattern could perhaps be labelled an “Obesogenic diet”.

It would be useful to look at free/added sugars in relation to a new method of classification of foods (NOVA system) that has become very popular based on how ingredients and foods are processed, especially regarding those labelled ultra-processed foods (UPF) [8]. This is not easy to relate to nutritional principles because nutrients are not part of the classification criteria. There are four food categories:

Unprocessed or minimally processed foods: fruit, vegetables, eggs, meat, milk and whole grains (these can in some cases be eaten raw but are very often eaten cooked).

Processed culinary ingredients usually consumed in combination with other ingredients: sugar, salt, butter, lard, oils and vinegar.

Processed foods: freshly made, unpackaged bread, tinned fruits and vegetables, salted nuts, ham, bacon, tinned fish and cheese.

Ultra-processed foods: these typically have five or more ingredients. They tend to include many additives and ingredients that are not typically used in home cooking and usually have a long self-life. They include ice cream, sausages, crisps, mass-produced bread, breakfast cereals, cookies, carbonated drinks, fruit-flavoured yogurts, instant soups and some alcoholic drinks, including whisky, gin and rum.

Free/added sugars are classed as “processed culinary ingredients” and once they have been added to other ingredients to form foods, as usually consumed, these foods are classed as UPF. This makes nutritional sense in most cases. However, for some foods the UPF classification appears somewhat arbitrary with regard to the current understanding of a healthy diet. As pointed out in the systematic review by Marino et al. [9], the UPF classification does not take into consideration the nutritional content of foods or their traditional and cultural importance within the diet of different populations. For example, the UPF classification is unrelated to the fibre content of the food, which is particularly important when considering bread and breakfast cereals. These foods are an important part of the diet in many Western countries and in their whole grain forms they are considered to be part of a healthy diet. For bread, a small amount of free sugars is necessary to activate the yeast and an automated production line is essential in order to feed large populations in towns and cities. These facts automatically lead to even a low-sugar, high-fibre wholegrain bread being classified as a UPF, thus implying that it is unhealthy. Likewise, with breakfast cereals, high-fibre, low-sugar versions are available and provide storable, ready-prepared foods which contain important nutrients but are classified as UPFs. In highly populated areas in these countries, limiting these foods could make providing affordable diets with adequate energy, nutrient and fibre content difficult, particularly when feeding under-privileged children.

An investigation into the relationship between UPF and free sugars intake has been carried out using data from NDNS in the UK, covering all age groups [10]. UPF accounted for 57% of total energy intake and 65% of free sugars intake overall. There was a linear increase in free sugars intake across quintiles of UPF consumption, except in the elderly. Reducing the intake of UPF would greatly reduce free sugars intake; however, this study did not look at the effects of this reduction on other nutrients.

If the UPF classification is applied to the foods cited above for the “obesogenic” dietary pattern [7]. Those that contribute to the high scores all fall into the UPF food category. However, at the opposite end of the spectrum, of the foods that are protective against obesity in this study, three were classed as unprocessed (fresh fruit, vegetables (not fried), potatoes (boiled)) and two as UPF (high-fibre breakfast cereals, high fibre bread). This is despite the fact that the fibre and nutrient content of the two UFP foods make an important contribution to the anti-obesogenic dietary pattern.

Some important work could be carried out by nutrition researchers investigating free/added sugars in the whole diet in different countries and settings:

(a) To replicate the finding that fat, fibre and free sugars intakes in combination are important for obesity development;
(b) To suggest ways of making the NOVA UPF classification more useful nutritionally and more aligned with tradition and culture in different countries.

These investigations into how foods and nutrients work together to improve health outcomes will help to clarify the nature of a healthy diet in different settings. This will prove important in communicating the healthy balanced diet message to the general public, who may be confused by the complicated UPF classification and how different versions of a healthy diet may be applied in their own lives.
